# Insight into the Role of the Aryl Hydrocarbon Receptor in Bovine Coronavirus Infection by an Integrated Approach Combining In Vitro and In Silico Methods

**DOI:** 10.3390/microorganisms13030579

**Published:** 2025-03-04

**Authors:** Luca Del Sorbo, Clementina Acconcia, Maria Michela Salvatore, Giovanna Fusco, Violetta Vasinioti, Maria Stella Lucente, Liqian Zhu, Annamaria Pratelli, Luigi Russo, Anna Andolfi, Rosa Iacovino, Filomena Fiorito

**Affiliations:** 1 Department of Veterinary Medicine and Animal Production, University of Naples Federico II, 80137 Naples, Italy; luca.delsorbo2@studenti.unina.it (L.D.S.); mariamichela.salvatore@unina.it (M.M.S.); 2 Department of Environmental, Biological and Pharmaceutical Sciences and Technologies, University of Campania Luigi Vanvitelli, 81100 Caserta, Italy; clementina.acconcia@unicampania.it (C.A.); luigi.russo2@unicampania.it (L.R.); 3 Istituto Zooprofilattico Sperimentale del Mezzogiorno, 80055 Portici, Italy; 4 Department of Veterinary Medicine, University of Bari, 70010 Valenzano, Italy; violetta.vasinioti@uniba.it (V.V.); mariastella.lucente@uniba.it (M.S.L.); annamaria.pratelli@uniba.it (A.P.); 5 College of Life Sciences, Hebei University, Baoding 071002, China; lzhu3596@163.com; 6 Department of Chemical Science, University of Naples Federico II, 80126 Naples, Italy; andolfi@unina.it

**Keywords:** bovine coronavirus, aryl hydrocarbon receptor signaling, CH223191

## Abstract

It is well known that the host response to different human and animal coronaviruses infection is regulated by the aryl hydrocarbon receptor, a ligand-activated transcription factor. The present study investigates the expression of the aryl hydrocarbon receptor during bovine coronavirus infection, through in vitro and in silico investigations. The in vitro studies demonstrate that the aryl hydrocarbon receptor and as well as its targets, CYP1A1 and CYP1B1, were significantly activated by bovine coronavirus infection in bovine cells (MDBK). During infection, the pretreatment of cells with non-cytotoxic doses of CH223191, a selective inhibitor of the aryl hydrocarbon receptor, resulted in a significant reduction in virus yield and a downregulation in the viral spike protein expression. These findings occurred in the presence of the inhibition of aryl hydrocarbon receptor signaling. Our results reveal that the bovine coronavirus acts on viral replication, upregulating the aryl hydrocarbon receptor and its downstream target proteins, CYP1A1 and CYP1B1. In addition, following the in silico studies, the three-dimensional structural model of the bovine aryl hydrocarbon receptor in complex with the antagonist CH223191 indicates that the molecular mechanism, by which the PASB and TAD domains of the receptor interact with the inhibitor, is mainly driven by an extensive network of hydrophobic interactions, with a series of hydrogen bonds contributing to stabilizing the complex. Interestingly, bioinformatic analyses revealed that the PASB and TAD domains in the human and bovine aryl hydrocarbon receptor present high similarity at the primary sequence and three-dimensional structure levels. Taken together, these findings represent a fundamental step for the development of innovative drugs targeting AhR as a potential object for CoVs therapy.

## 1. Introduction

*Coronaviridae* (CoVs) are enveloped single-stranded RNA viruses, generally responsible for upper and lower respiratory infections and gastrointestinal disorders both in mammalians and birds [1,2]. The four genera, *Alphacoronavirus* (αCoV), *Betacoronavirus* (βCoV), *Gammacoronavirus* (γCoV), and *Deltacoronavirus* (δCoV), included in the subfamily *Orthocoronavirinae* are characterized by a broad tissue tropism and by the ability to overcome interspecies barriers [1,2]. For example, human coronaviruses (HCoVs), which until 2003 were causative agents only of the common cold or enteritis, often have animal origin, as result of an adaptation to humans by jumping directly or through an intermediate animal host [3,4]. As a matter of fact, genetic data have demonstrated that some human αCoVs, such as HCoV-OC43 and HECV-4408, are the results of a host jump from bovine coronavirus (BCoV), which derived from a bat virus through adaptation in a rodent [5,6,7]. In the last decades, the emergence of severe acute respiratory syndrome coronavirus 1 (SARS-CoV-1), Middle East respiratory syndrome coronavirus (MERS-CoV), and SARS-CoV-2 highlight the role and importance of public health with respect to CoVs, which for many years were of exclusive interest to veterinary medicine. Immediately after SARS-CoV-2 was declared a pandemic by the World Health Organization (WHO) on 11 March 2020 [8], global scientific interest focused on the study of viral pathogenesis, vaccine development, and the search for effective drugs to counteract the spread of the infection.

Although the use of vaccines has had a decisive and favorable impact on the control of the pandemic, the development of effective antiviral compounds remains critical [9,10,11,12]. Research on antiviral therapy is a fundamental objective to counteract the virus due to the current emergency and spread of variants that escape immunity induced by vaccines prepared with old strains even in immunocompetent people as well as for the protection of immunocompromised persons who are unlikely to respond effectively to vaccination [13]. Specific compounds targeting conserved CoV proteins could also prove effective against future pandemic CoV infections.

**Figure 1:**
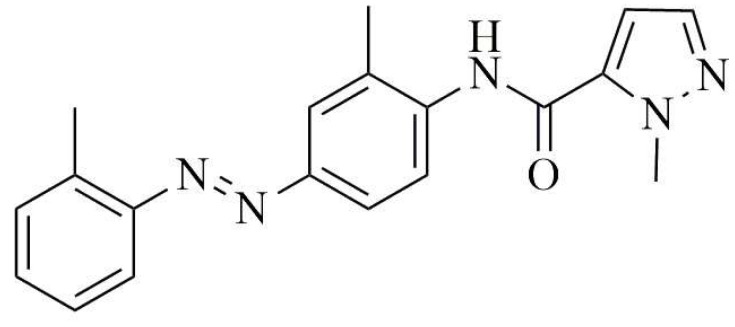
Chemical structure of the AhR inhibitor CH223191.

Antiviral drugs, in general, aim to act on specific enzymes involved in the replication mechanism of viruses or in the assembly phase, and different compounds have been shown to counteract and treat SARS-CoV-2 viral disease [11,12]. In CoV infection, the aryl hydrocarbon receptor (AhR), a transcription factor activated by endogenous and exogenous substrates, modifies natural immune responses of host, acting on cytokine levels [14]. For instance, AhR activation has been reported during infection with mouse hepatitis virus (MHV), MERS-CoV, HCoV229E, SARS-CoV-1, SARS-CoV-2, canine coronavirus (CCoV), porcine epidemic diarrhea virus (PEDV), and feline coronavirus (FCoV) [15,16,17,18,19,20,21,22]. Recently, Giovannoni et al. [16] demonstrated that AhR is activated by infection with SARS-CoV-2, and pharmacological inhibition of AhR can suppress in vitro replication of both SARS-CoV-2 and HCoV 229E. Therefore, it is possible to suppose that AhR may represent a potential target for antivirals, enhancing the host response to CoVs infection. In this context, to avoid the risks deriving from the use of a highly pathogenic and contagious virus, the involvement of AhR response during infection with BCoV, a βCoV-like SARS-CoV-2, and the activity of a selective AhR ligand (CH223191, Figure 1) were evaluated by in vitro studies and by the application of an integrated approach based on the combination of computational techniques such as homology modeling and molecular docking.

## 2. Materials and Methods

### 2.1. Cell Cultures and Virus Infection

MDBK cells were cultured in Dulbecco’s modified Eagle’s minimal essential medium (DMEM) supplemented with 10% fetal bovine serum (FBS) and incubated at 37 °C and 5% CO_2_ [23,24]. BCoV, strain 282/23, stored by the Sector of Infectious diseases of the Department of Veterinary Medicine (University of Bari Aldo Moro, Bari, Italy), was used throughout the study and was cultured and titrated in MDBK cells.

2-Methyl-2H-pyrazole-3-carboxylic acid (2-methyl-4-o-tolylazo-phenyl)-amide (CH223191) (Sigma-Aldrich, St. Louis, MI, USA) is a highly specific AhR competitive antagonist [25,26] and was solubilized in dimethyl sulfoxide (DMSO) (Sigma-Aldrich) at concentrations of 2, 5, 10, and 20 µM [16,17]. MDBK cells were pretreated for 1 h at 37 °C with DMEM supplemented with 10% FBS containing CH223191 (2, 5, 10, and 20 µM). Cells were then infected or not with BCoV, at a multiplicity of infection (MOI) of 0.05 and 0.5 to obtain four groups: (a) untreated uninfected cells; (b) untreated infected cells; (c) CH223191-treated uninfected cells; and (d) CH223191-treated infected cells. After 1 h of adsorption at 37 °C, cells were incubated and processed at 24 h post infection (p.i.). BCoV remained in the culture medium for the entire experiment.

### 2.2. Cell Viability

Cell viability was assessed by the Trypan Blue (TB) (Sigma-Aldrich) exclusion test as previously reported [27,28,29]. MDBK cell monolayers, pre-treated or not with CH223191 at different concentrations (2, 5, 10, 20, and 50 µM), were infected or not with BCoV at a MOI of 0.05, and at 24 h post infection (p.i.) cells were trypsinized, mixed with TB, and then counted through a TC20 automated cell counter (Bio-Rad Laboratories, Hercules, CA, USA). The number of living cells over the total cell number was determined as the percentage, and the results were reported as the mean ± S.D. of three independent experiments performed twice. Furthermore, cell viability was determined by using Trypan-blue in cells attached to wells, as previously reported [27,29]. Cell viability (IC_50_) calculation in MDBK cells was evaluated by IC_50_ Calculator|AAT Bioquest, Inc., Pleasanton, CA, USA (https://www.aatbio.com/tools/ic50-calculator, accessed on 8 January 2025).

### 2.3. Examination of Cell Morphology

Cell monolayers, pretreated or not with CH223191, were infected or not with BCoV (MOI 0.05) and incubated for 24 h. After that, cells were washed with PBS and stained with Giemsa and acridine orange/propidium iodide (AO/PI) [30,31,32], and morphological features of cell death were observed [33,34,35].

For Giemsa staining, cells were fixed with 95% ethanol, drained, dried, and stained with a 5% Giemsa solution (Merck, Darmstadt, Germany) for 30 min. Then, cells were rinsed with tap water and H_2_O and observed at light microscopy using a ZOE Cell Imager (Bio-Rad Laboratories). After staining cells with AO/PI, fluorescence microscopy was assessed by a ZOE Cell Imager (Bio-Rad Laboratories) to identify viable and dead cells [32]. Indeed, green fluorescence is provoked by the binding of AO (membrane-permeability) to nucleic acids; the PI impermeability to intact cell membrane induces the cross of dead and dying cells, intercalating nucleic acids that produces a bright red fluorescent complex.

### 2.4. Immunofluorescence Staining

MDBK cells, pretreated or not with CH223191, were infected or not with BCoV, at MOI 0.5. Immunofluorescence staining was assessed at 24 h p.i. [29,36] by the following antibodies and antisera, diluted in 5% bovine serum albumin-1x Tris-Buffered Saline, 0.1% Tween^®^ 20 Detergent: (a) anti-aryl hydrocarbon receptor (AhR) (Sigma-Aldrich, St. Louis, MI, USA) (1:250); (b) mouse anti-Bovine Coronavirus Spike Antibody (5A4) (MAB12430, The Native Antigen Company, Oxford, UK) (1:400); (c) mouse monoclonal anti-CYP1A1 (A-9) (sc-393979, Santa Cruz Biotechnology, Inc., Dallas, TX, USA); (d) mouse monoclonal anti-CYP1B1 (G-4) (sc-374228, Santa Cruz Biotechnology, Inc.); (e) goat anti-rabbit Texas Red (Thermo Fisher Scientific, Waltham, MA, USA) (1:100); (f) goat anti-mouse Alexa Fluor 488 (Thermo Fisher Scientific) (1:1000). In the following sections, the fluorescence signals from microscopy images, assessed by ZOE Fluorescent Cell Imager (Bio-Rad Laboratories), were determined by ImageJ (National Institutes of Health) software (Java 1.8.0_345). The fluorescence intensity was determined and plotted versus the control group (DMSO). The results of one experiment, representative of three independent experiments, was reported for each figure.

### 2.5. Virus Production

MDBK cells, pretreated or not with CH223191, were infected or not with BCoV, at an MOI of 0.5, incubated at 37 °C, and processed at 24 h of infection. After three freezing and thawing cycles, cells were aliquoted and stored at –80 °C, and virus titration was assessed by the TCID_50_ method, according to Reed and Muench (1938), as previously reported [37]. Moreover, the cytopathic effect (CPE) was evaluated at 24 h p.i. after fixation with methanol and staining with crystal violet [24].

### 2.6. Statistical Analysis

Results are expressed as mean ± S.D. One-way ANOVA with Tukey’s post test and by Student’s *t* test was assessed by GraphPad InStat Version 3.00 for Windows 95 (GraphPad Software, San Diego, CA, USA). *p* < 0.05 was statistically significant.

### 2.7. Homology Modeling and Molecular Docking Studies

Because the 3D structure of the bovine aryl hydrocarbon receptor (bAhR) was not available in the protein data bank (PDB), we built the three-dimensional structural model of bAhR using AlphaFold 3.0. The latter software employs advanced artificial intelligence and machine learning algorithms to generate highly accurate predictions of protein structures based on their amino acid sequences [38]. The modelling process was carried out using the folded region of the receptor, consisting of residues from 1 to 400, with default parameters. In detail, the calculation was launched with an MMseqs2 option for multiple sequence alignment (MSA) searching, an unpaired mode for generating separate MSA for each protein and no filter options for pair_cov (minimum coverage with query (%)), and pair_qid (minimum sequence identity with query (%)). The structural 3D models were obtained using the following parameters: number of models = 5; max recycles = 3. The calculated conformers were very similar, and the one with the highest rank based on pLDDT was selected as a representative structure and was used for further docking studies. In addition, the quality of the selected representative structural model of bAhR was assessed by evaluating the Ramachandran plot obtained using the software PROCHECK v. 3.5 (SI1). After that, to describe the molecular determinants driving the formation of the bAhR/CH223191 complex, the selected 3D Alpha Fold structural model of bAhR was used for molecular docking studies. To dock the ligand CH223191 to bAhR, we used the software Dockthor v. 1.0 [39], which uses a specialized force field that incorporates Van der Waals forces, electrostatic interactions, and implicit solvent effects, leading to higher accuracy in predicting protein–ligand interactions.

The molecular docking calculation involved the following steps: (1) preparation of the starting coordination files for the protein and the ligand in order to include in the protocol the required information, such as spatial charges and torsional degrees of freedom. For bAhR, the protonation states of side chains were assigned according to physiological pH using the Dockthor platform built-in preparation tools [39]. For CH223191, the three-dimensional structure was built using the Dockthor ligand preparation module, by which missing hydrogen atoms were added and the geometry of the ligand was optimized through energy minimization by applying the MMFF94 force field, and then, the PDBQT format, including partial and rotatable charges, was generated; (2) definition of the docking grid surrounding the active site of the AhR receptor. Grid dimensions were chosen to include key residues implicated in ligand binding, as identified in 400,221 total points. The docking parameters were carefully configured to ensure accurate and reliable results. The grid box dimensions were set to adequately cover the binding pocket of the receptor, with specific values chosen along the X, Y, and Z axes to encompass the critical areas of interaction. The docking simulation was executed using Dockthor, generating a set of potential binding conformations ranked by their predicted binding free energies. Additionally, the number of binding poses generated was selected to thoroughly explore the various orientations and conformations that the ligand could adopt within the receptor site; (3) selection of the top-ranking pose for the bAhR/CH223191 complex by evaluating the binding energy. The 3D docking model with the lowest binding energy score was selected as representative conformer, and it was analyzed and visualized by PyMoL v. 3.1 [40] and Chimera v. 1.18 [41].

### 2.8. Validation of the Docking Model of the bAhR/CH223191 Complex Obtained by Dockthor

We evaluated the accuracy and the stability of the three-dimensional structural model of bAhR receptor bound to CH223191 obtained using Dockthor v. 1.0 program by applying a two-step validation strategy: (i) We applied a different docking protocol using the AutoDock 4.0 program. In detail, after preparing the starting coordinate files for the ligand and the receptor, we pre-calculated the required grid maps using the AutoGrid tool. The grid map consists of a 3D lattice of regularly spaced points, entirely or partly surrounding and centered on a specific region of the receptor. In our validation docking protocol, the grid size was set to be 40 × 50 × 40, and the grid space was 0.375 Å. After that, we started the docking procedure using the AutoDock routine: a docking file, containing the input parameters for the calculation, was created using AutoDockTools. In detail, we used as a searching method the Lamarckian Generic Algorithm (LGA). Minimized ligands were randomly positioned inside the grid box, and the docking process initiated with a quaternion and torsion steps of 4 torsional degrees of freedom, the number of energy evaluations at 2,500,000, and a run number of 100. After the docking simulation, the bAhR/CH223191 structures were clustered using a backbone RMSD cutoff of 2 Å. The clustering analysis generated three clusters containing 96, 2, and 2 structures. The most populated cluster (cluster 1), having the lowest binding energy, was used to select the representative conformer. (ii) We predicted the binding energy using PRODIGY-LIG v. 1.1.0 software [42,43], which is an effective predictive model based on intermolecular contacts of the 3D structure of protein–protein and protein–ligand complexes. This method is able to predict the binding free energy between protein complexes with great accuracy.

## 3. Results

### 3.1. CH223191 Increases Cell Viability During BCoV Infection

**Figure 2:**
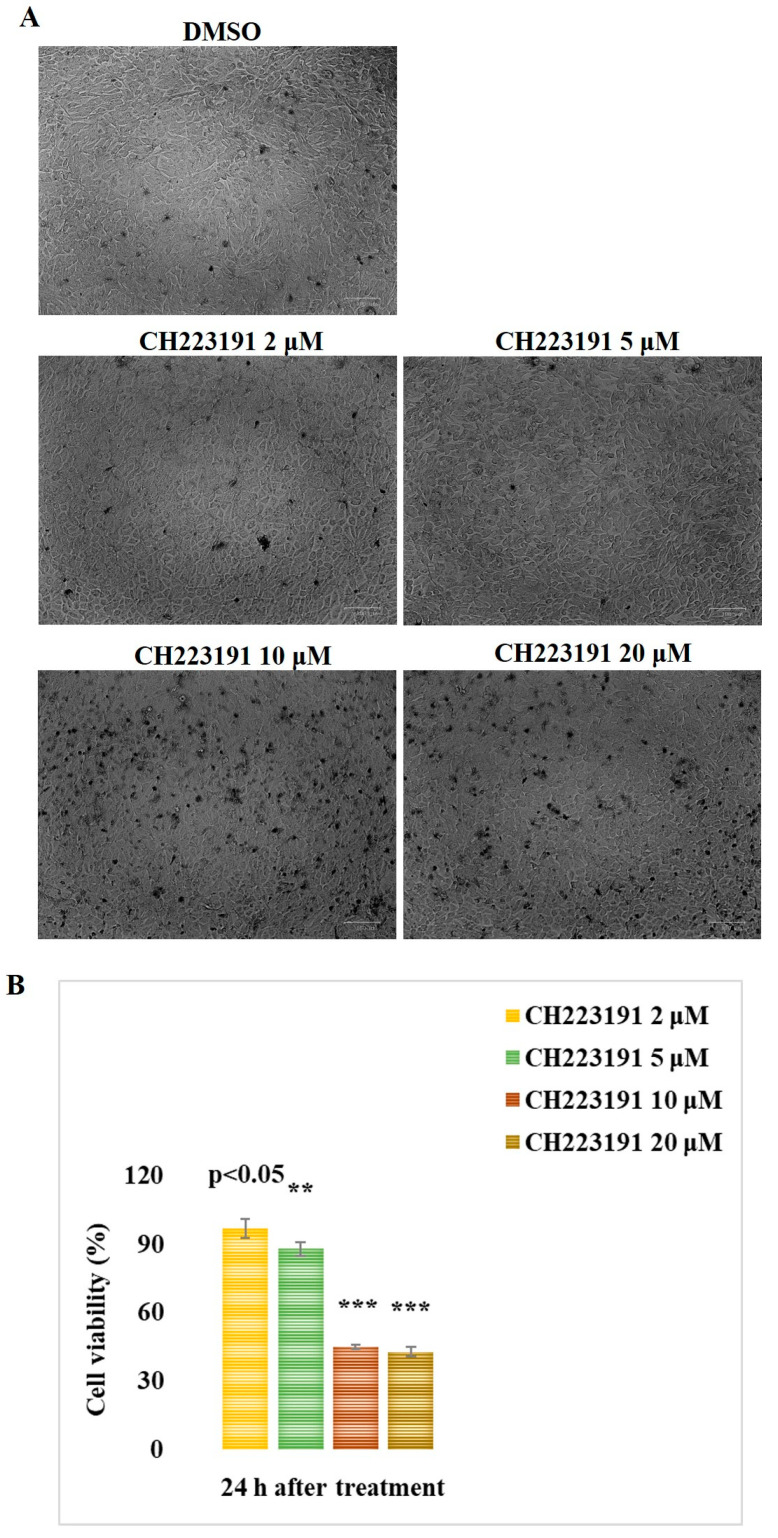
The AhR inhibitor CH223191 at the concentration of 2 µM induces no significant (*p* > 0.5) differences in MDBK cell viability after 24 h of pretreatment. (**A**) Microscopic MDBK cells treated with DMSO or with CH223191 at different concentrations and stained with TB while cells were attached to wells. Scale bar 100 µm. (**B**). Identification of the IC_50_ of CH223191 inhibitor by using different concentrations (2, 5, 10, and 20 μM) and development of dose–response curve in MDBK cells after 24 h of pretreatment. Cell viability was assessed by TB staining and scored by an automated cell counter. Significant differences between DMSO and CH223191-treated cells are indicated by probability *p*. ** *p* < 0.01 and *** *p* < 0.001.

The effects of the AhR inhibitor (CH223191) at the concentrations of 2, 5, 10, and 20 μM were tested on uninfected MDBK cells after 24 h of treatment. A dose–response curve was produced to calculate the cytotoxic concentration of the inhibitor CH223191 reducing the cell viability by 50% (IC_50_) in MDBK cells (Figure 2). Cell viability (% of control) was calculated at 24 h of treatment, with a IC_50_ of 6.2893 μM CH223191 (Figure 2). No significant differences in MDBK cell viability (*p* > 0.5) were induced by the concentration of 2 µM CH223191 (Figure 2), which was then employed for further investigations.

**Figure 3:**
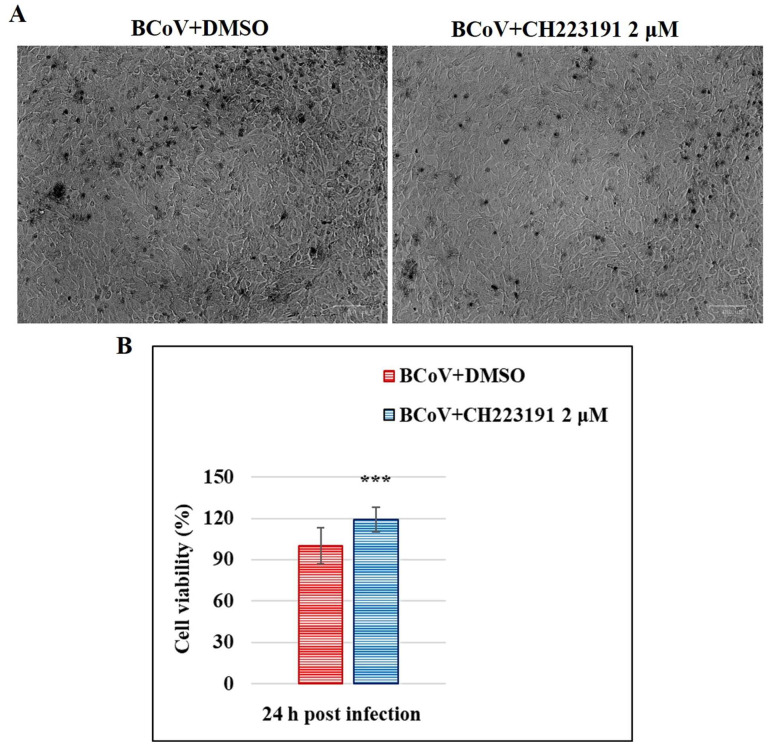
AhR inhibitor CH223191 increases cell viability during BCoV infection. (**A**) MDBK cells pretreated or not with CH223191 at 2 µM and infected with BCoV. At 24 h p.i., cells were stained with TB while cells were attached to wells and observed under a light microscope. Scale bar = 100 µm. (**B**) Dose–response curve of MDBK cells pretreated with CH223191 at 2 μM and infected with BCoV. After 24 h of infection, cell viability was determined by TB staining and scored by automated cell counter. Significant differences between BCoV+DMSO and BCoV+CH223191-treated cells are indicated by probability *p*. *** *p* < 0.001.

Subsequently, MDBK monolayers were pretreated or not with CH223191 (2 μM) and infected with BCoV at an MOI of 0.05. After infection (24 h p.i.), the group pre-treated with CH223191 at 2 µM induced a significant (*p* < 0.001) increase in MDBK cell viability (Figure 3).

### 3.2. CH223191 Reduces Morphological Cell Death Features During BCoV Infection in MDBK Cells

**Figure 4:**
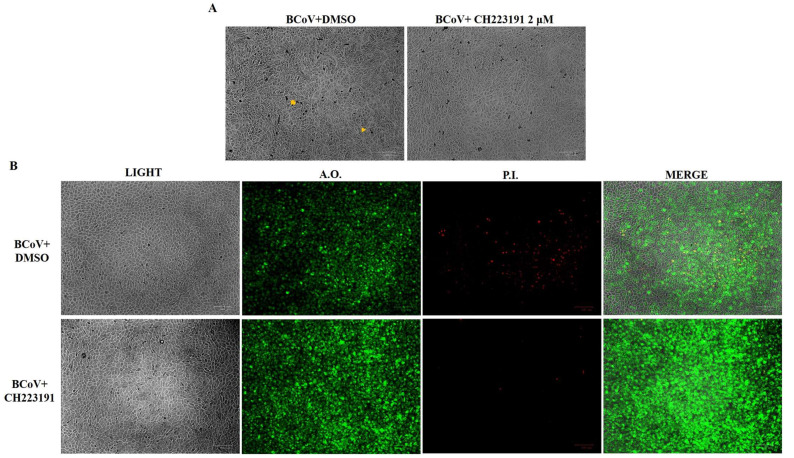
AhR inhibitor CH223191 reduced morphological signs of cell death during BCoV infection in MDBK cells. Cells pretreated or not with CH223191 were infected with BCoV. At 24 h p.i., cells were stained with (**A**) Giemsa and analyzed under a light microscope. Morphological features of cell death, such as cellular shrinkage (arrowhead) and pyknosis and chromatin condensation (arrow) were mainly reduced in the CH223191-treated infected groups. (**B**) In AO/PI panels, PI fluorescent cells, indicating dead and/or dying cells, were mainly detected in BCoV-infected cells compared to CH213191-treated infected cells. Scale bar 100 µm. The results of one experiment representative of three independent experiments were reported.

Giemsa staining revealed the morphological signs of cell death. In infected and DMSO-treated cells, shrinkage (Figure 4A, arrowhead), pyknosis, and chromatin condensation (Figure 4A, arrow) were observed. These cell death features were reduced in BCoV-infected cells pretreated with CH223191 (Figure 4). In AO/PI staining, MDBK cells were observed under fluorescence microscopy to detect viable and dead cells. The AhR inhibitor induced a relevant decrease in PI fluorescence in BCoV-infected groups compared to infected-untreated cells (Figure 4B).

These results demonstrated that the AhR inhibitor CH223191 considerably preserved the viability of bovine cells during BCoV infection.

### 3.3. AhR Inhibitor Decreases Virus Yield During BCoV Infection CPE Evaluation

**Figure 5:**
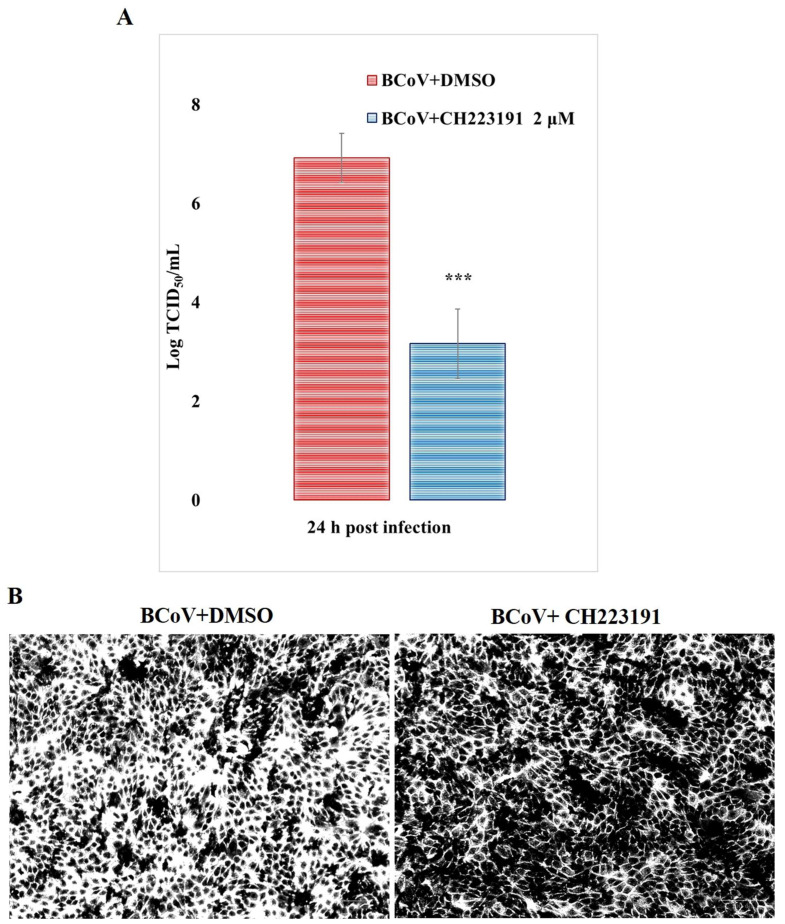
AhR inhibitor CH223191 induces a reduction in virus yield during BCoV infection in MDBK cells. Cells pretreated or not with AhR inhibitor CH223191 were infected with BCoV at 24 h p.i. (**A**) Virus yield was assessed by the TCID_50_ method and reported as Log TCID_50_/mL. Significant differences between BCoV-infected cells and CH223191-treated infected cells are indicated by probability *p*. *** *p* < 0.001. (**B**) CPE by crystal violet staining was detected by the ZOE Cell Imager. Scale bar 100 µm. The results of one experiment representative of three independent experiments were reported.

During BCoV infection in MDBK cells, the pretreatment with CH223191 affected virus production. Indeed, BCoV virus titer (expressed in Log) at 24 h p.i. was significantly reduced (*p* < 0.001) in cells pretreated with CH223191 (Figure 5A) compared to BCoV + DMSO control groups.

After crystal violet staining, microscopic analysis of infected cells showed an increase in CPE in untreated cells compared to the CH223191-treated group (Figure 5B).

Overall, our results demonstrated that the AhR inhibitor CH223191 induced a significant reduction in BCoV yield during infection in MDBK cells.

### 3.4. AhR Was Expressed in MDBK Cells

**Figure 6:**
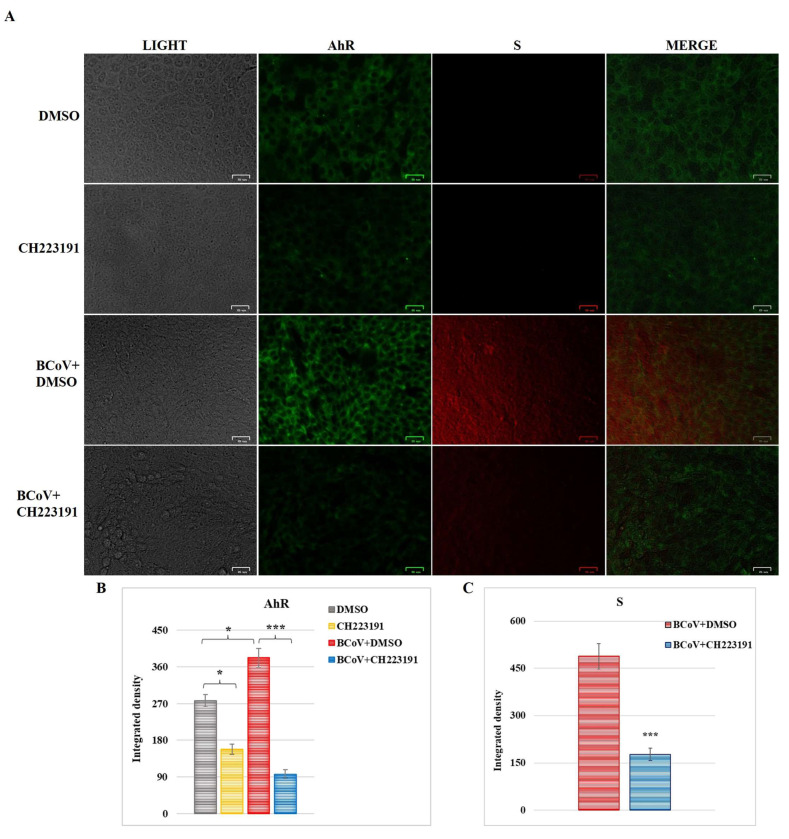
AhR is expressed in MDBK cells. AhR inhibitor CH223191 significantly induced a reduction in AhR expression in MDBK cells. BCoV activates the expression of AhR, and the AhR inhibitor (CH223191) downregulates both AhR and S protein expression during BCoV infection in MDBK cells. (**A**) In CH223191-treated and untreated uninfected cells, as well as in CH223191-treated and untreated BCoV-infected cells, immunofluorescence staining was performed to assess AhR and S protein expression. Scale bar = 25 µm. (**B**,**C**) Bars are the mean ratio generated from the integrated density (product of area and mean fluorescence intensity) of the AhR and S protein expression during BCoV infection. Significant differences between control (DMSO-treated) and BCoV-infected cells, as well as between BCoV-infected cells and AhR-inhibitor-treated infected cells for both AhR and S proteins, are indicated by probability *p*. * *p* < 0.05 and *** *p* < 0.001. The integrated density was measured by ImageJ. Error bars represent standard deviation measurement. The results of one experiment representative of three independent experiments were reported.

Immunofluorescence staining was used to detect the expression of AhR in MDBK cells (Figure 6A). AhR expression in bovine cells was strongly reduced by the inhibitor CH223191 (Figure 6A,B).

### 3.5. BCoV Infection Activates the Expression of AhR

During BCoV infection, the expression of AhR was significantly increased in MDBK cells (Figure 6A). This result was demonstrated by the calculation of integrated density, indicating an increase in AhR induced by BCoV infection, which was 1.37-fold higher than in uninfected MDBK cells (Figure 6B).

### 3.6. Inhibitor CH223191 Inhibits Both AhR and S Expression During BCoV Infection

The expression of AhR and S proteins was examined during BCoV infection in MDBK cells (Figure 6). The level of both AhR and S proteins was drastically reduced in CH223191-infected groups compared to untreated-infected cells (Figure 6A). These results were corroborated by integrated density fluorescence measurement (Figure 6B) and suggest that BCoV infection activated AhR expression, and that the pretreatment with the AhR inhibitor CH223191 leads to a remarkable reduction in the expression of both AhR and S proteins.

Our results demonstrated that the AhR, expressed in MDBK cells, was upregulated by BCoV infection, and the AhR inhibitor CH223191 induced a significant reduction in both AhR and S expression during infection in MDBK cells.

### 3.7. BCoV Infection Activates the Expression of Both CYP1A1 and CYP1B1 Proteins

**Figure 7:**
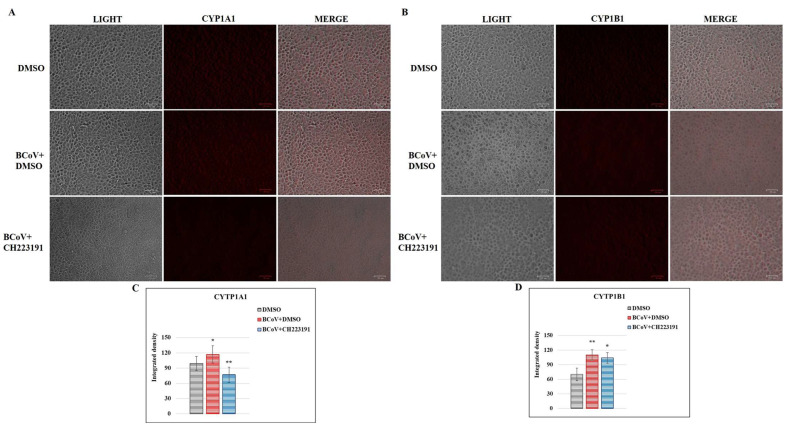
BCoV activates the expression of both CYP1A1 and CYP1B1 (AhR signaling) during infection in MDBK cells. MDBK cells, pretreated or not with AhR inhibitor, were infected with BCoV at an MOI of 0.5 for 24 h. Then, immunofluorescence staining with antibodies recognizing (**A**) CYP1A1 and (**B**) CYP1B1 was performed. Scale bar = 50 µm. (**C**,**D**) Bars are the mean ratio generated from the integrated density (product of the area and mean fluorescence intensity) of the CYP1A1 and CYP1B1 expression during BCoV infection. Significant differences between DMSO and BCoV-infected cells, as well as between BCoV-infected cells and AhR-inhibitor-treated infected cells for both CYP1A1 and CYP1B1 proteins, are indicated by probability *p*. * *p* < 0.05 and *p*. ** *p* < 0.01. The integrated density was measured by ImageJ. Error bars represent standard deviation measurement. The results of one experiment representative of three independent experiments were reported.

The expression of AhR signaling, by assessing cytochromes CYP1A1 and CYP1B1, was evaluated in MDBK cells during BCoV infection (Figure 7A,B). A significant increase in the expression of both cytochromes was observed in the infected cell groups, while pretreatment with CH223191 led to a marked down-regulation of their expression in infected cells (Figure 7C,D).

### 3.8. Structural Model of the bAhR in Complex with CH223191

**Figure 8:**
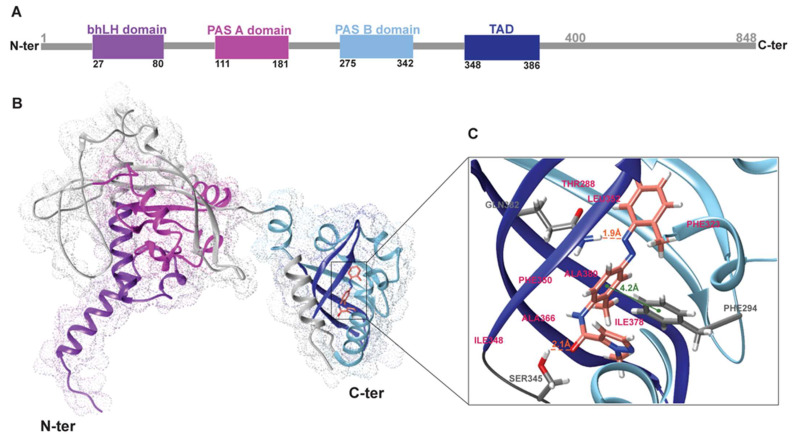
Recognition mechanism of CH223191 at the bAhR. (**A**) Structural representation of the full-length b AhR r, highlighting its four key domains: the bHLH (basic Helix-Loop-Helix) domain, spanning residues 27–80; the PAS A (Per Arnt Sim A) domain, spanning residues 111–181; the PAS B (Per Arnt Sim B) domain, spanning residues 275–342; and the TAD (Transactivation Domain), spanning residues 348–386. Each domain plays a crucial role in the function of the receptor and ligand interaction, providing a detailed understanding of the structural organization of the receptor. (**B**) 3D model of the bAhR (residues 1–400) predicted by AlphaFold, showing the folded regions containing the bHLH, PAS A, and PAS B domains. (**C**) Docking model of the CH223191 ligand bound to the bAhR. The figure highlights two key hydrogen bonds formed with residues Gln382 and Ser345, a π–π interaction with the aromatic side chain of Phe294, and several hydrophobic interactions with surrounding residues of the receptor, all of which are illustrated in the figure. These interactions contribute to the stable binding of CH223191 within the ligand-binding domain of the receptor.

In order to describe the molecular mechanism by which the bAhR recognizes the CH223191 ligand, we applied a computational approach integrating homology modeling methodologies with molecular docking methods. First, the three-dimensional structure of the N-terminal portion of the AhR, encompassing the region from ASN1 to THR400 (AhR1-400), was predicted using the software AlphaFold 3.0, and its quality was assessed by analyzing the Ramachandran plot. As reported in Figure 8A,B, the obtained 3D structural model of AhR1-400 indicates the presence of the four functional domains: the basic helix-loop-helix (bHLH) domain, which is essential for DNA binding and receptor dimerization; the PAS-A and PAS-B domains, which play crucial roles in protein–protein interactions and ligand binding; and the C-terminal transactivation domain, which regulates transcriptional responses. To note, the Ramachanidran plot shows a remarkable quality of the predicted model, with more than 99% residues within energetically allowed regions for the φ and ψ torsion angles (most favored and additional allowed regions) (Figure S1). Successively, the CH223191 ligand was docked on the obtained structure of the bovine AhR1-400 receptor by Dockthor. Then, we evaluated the accuracy and stability of the Dockthor structural model by using a two-step validation strategy.

First, we predicted the structure of the AhR1-400/CH223191 complex by applying a different docking protocol using Autodock 4.0 software [44]. As a result, the docking calculation generated 100 conformers that, as reported in the materials and methods, were sorted into clusters. This procedure resulted in three clusters (Figures S2A–D), characterized by a different binding energy and showing small but significant differences in the terms of the relative ligand orientation within the binding pocket. To note, the most populated cluster (Cluster 1), composed of 96 conformers, showed the lowest binding energy. Therefore, we compared the structure having the lowest binding energy within cluster 1 with the structure obtained by Dockthor. As reported in Figure S2E, the ligand CH223191 shows, in the two structural docking models, an identical binding pose demonstrating that the Dockthor structural model provides an accurate description of the AhR1-400/CH223191 complex.

Second, we evaluated the stability of the bAhR/CH223191 complex by estimating the binding energy using the PRODIGY web server, which is able to produce results comparable with those obtained experimentally [42]. The binding energy obtained for the Dockthor docking structural model was −5.33 kcal/mol, suggesting that the complex is reasonably stable. Overall, our structural analysis demonstrates that the structural model of the AhR1-400/CH223191 complex obtained by Dockthor reports a rigorous characterization of the molecular determinants governing the recognition mechanism of AhR by CH223191.

The 3D structural model of the AhR1-400/CH223191 complex indicates that the molecular mechanism by which the receptor binds the ligand is mainly driven by an extensive network of hydrophobic interactions involving Thr288, Phe294, Leu307, Leu314, Phe323, Ile348, Phe350, Leu352, Ala366, Ile378, and Ala380. In addition, the binding of CH223191 to the receptor is further stabilized by a series of hydrogen bonds. In detail, the ligand makes hydrogen bond interactions with Ser345 and Gln382, suggesting the important role of these receptor residues in modulating the recognition mechanism in terms of binding affinity and of the relative ligand orientation within the binding pocket of the receptor. Furthermore, π-stacking interactions between CH223191 and PHE294 enhance the receptor/ligand complex (Table S1). These findings, describing the structural determinates involved in the binding of the antagonist CH223191 to the bovine AhR receptor, may have significant implications for further studies on receptor modulation and therapeutic targeting of AhR-related pathways.

## 4. Discussion

Coronaviruses are relevant viruses, taking as an example the severe acute pandemic respiratory syndrome (COVID-19), which highlights how little we know about these viruses. Emerging viral diseases, as a matter of common knowledge, have very few, if any, effective treatment options and often purpose old antiviral drugs. Hence, the exploration of new active molecules represents considerable challenges arising from justified concerns for disease control. Besides vaccinations, the drugs currently approved for SARS-CoV-2 to treat COVID-19 by the FDA only include a few molecules, like nirmatrelvir and ritonavir, in combination (for infected adults but being treated outside of the hospital), remdesivir (for certain adult and pediatric patients, both hospitalized or not), baricitinib, and tocilizumab in certain hospitalized adults [45]. All these FDA-approved compounds are critical actors in the viral cycle of SARS-CoV-2, because they act as inhibitors of the RNA-dependent RNA polymerase (RdRp), 3C-like protease (3CLpro), as well as the serine-threonine protein kinases, named adaptor-associated kinase 1 and cyclin G-associated kinase, which are involved in SARS-CoV-2 entry via endocytosis. For instance, nirmatrelvir acts as an effective inhibitor of the main protease of SARS-CoV-2 but is quickly metabolized by the cytochrome CYP3A4 enzyme in vivo. The bioavailability of nirmatrelvir, for therapeutic use, is enhanced by ritonavir, which inhibits the CYP3A4 enzyme [11,12,46]. Remdesivir also targets RdRp, but its promising therapeutic efficacy is regrettably debated, due to a drug-resistance phenomenon in patients with low immune system defense [12].

Taking into account what was previously reported, the main target of this work was to investigate the role of AhR during BCoV infection to expand the knowledge on the mechanism of action of a βCoV-like SARS-CoV-2, which may represent an excellent model for the study of the pandemic CoV. Consequently, BCoV infection in cattle can be used as an animal model for the study of new therapeutic strategies against SARS-CoV-2 infection in humans. To achieve this goal, in vitro and in silico studies were developed to elucidate the role of AhR during BCoV infection. The involvement of AhR, a receptor expressed in several mammalian cells [14], during some α- and β-CoV infections has been reported in previous studies [15,16,17,18,19,20,21,22]. However, here for the first time, following infection in bovine (MDBK) cells, the activation of AhR was observed during BCoV infection (Figure 6). The pretreatment with a non-toxic concentration of CH223191 (2 μM), a specific AhR inhibitor, to infected cells turned out to be efficient enough to inhibit the expression of AhR as well as of viral protein S. These findings occurred together with a significant decrease in both virus yield and cell viability during BCoV infection. In addition, in the presence of CH223191, an improvement of morphological features in MDBK-infected groups was also detected. Furthermore, the infection led to the upregulation of CYP1A1 and CYP1B1 levels (Figure 7), which are downstream target proteins in AhR signaling. A similar trend was also reported for other CoV infections [16,18,19,21,47].

Interestingly, the pre-treatment with CH223191 induced a decrease in the expressions of the tested cytochromes. Our results showed that AhR signaling was modulated during BCoV infection in vitro, demonstrating a pivotal role of this receptor in another CoV. The combined in silico approach provided comprehensive insights into the molecular interactions between CH223191 and bAhR. In particular, by using molecular modeling techniques, the structural model of bAhR (residues 1–400), was successfully predicted with a high degree of precision, as revealed by Ramachandran plot analysis. The 3D structural model of AhR^1−400^ indicates the presence of the four functional domains including the basic helix-loop-helix (bHLH) domain, the PAS-A and PAS-B domains, and the transactivation domain (TAD) [48,49]. These regions are essential for receptor functions such as DNA binding, receptor dimerization, and ligand interaction. Yet, according to previous studies [50], our validated molecular docking studies demonstrate that the bAhR recognition mechanism by the inhibitor CH223191 is driven by an extensive network of interactions, comprising hydrophobic contacts and hydrogen bonds, which play a crucial role in the formation and stabilization of the CH223191-bAhR complex. This latter is characterized by reasonable stability, as revealed by the estimated binding energy (−5.33 kcal/mol). Overall, our structural data elucidate the molecular basis involved in the inhibitory mechanism of CH223191, which likely interferes with the ability of the receptor to propagate downstream signaling pathways essential for viral replication.

Taken together, in vitro and in silico results showed that BCoV, a βCoV-like SARS-CoV-2, activates the AhR pathway during infection. Following infection in mammalian cells (MDBK), the pre-treatment with a well-known AhR inhibitor, such as CH223191, downregulates AhR signaling, provoking a substantial reduction in virus yield. Our findings also demonstrate the high specificity of CH223191 for bAhR and highlight the potential of this receptor as a target of therapeutic strategies suitable to fight CoVs infection.

## Data Availability

The original contributions presented in this study are included in the article/Supplementary Material. Further inquiries can be directed to the corresponding authors.

## Supplementary Materials

The following supporting information can be downloaded at: https://www.mdpi.com/article/10.3390/microorganisms13030579/s1, Table S1. Schematic representation of the residues of the bovine AhR receptor involved in interactions with the inhibitor CH223191, as determined by docking calculations. The table lists the specific residues and their corresponding interactions, including hydrogen bonds, hydrophobic contacts, and other interactions that contribute to the binding affinity of CH223191 to the receptor. Figure S1. Structural analysis of the three-dimensional model of the bovine Aryl Hydrocarbon Receptor (bAhR). The Ramachandran plot illustrates the conformational angles of the structural model, which was generated using AlphaFold, highlighting the distribution of dihedral angles and assessing the stereochemical quality of the predicted protein structure. Figure S2. Molecular docking studies of the bovine Aryl hydrocarbon receptor (bAhR) obtained by the AutoDock 4.0 program.
