# Supplementary material for: Insight into the Role of the Aryl Hydrocarbon Receptor in Bovine Coronavirus Infection by an Integrated Approach Combining In Vitro and In Silico Methods
Source: Microorganisms. 2025 Mar 4;13(3):579. doi: 10.3390/microorganisms13030579 (PMC11944835; doi:10.3390/microorganisms13030579)
Supplement: Supplementary file 1 [file microorganisms-13-00579-s001.zip › microorganisms-3442983-supplementary-final-author checked (1).pdf]

Supplementary Information

Insight into the Role of the Aryl Hydrocarbon Receptor in Bovine Coronavirus Infection by an Integrated Approach Combining In Vitro and In Silico Methods

Luca Del Sorbo[1,$], Clementina Acconcia[2,$], Maria Michela Salvatore[1,$], Giovanna Fusco[3,*], Violetta Vasinioti[4], Maria Stella Lucente[4], Liqian Zhu[5], Annamaria Pratelli[4], Luigi Russo[2], Anna Andolfi[6], Rosa Iacovino [2,*], Filomena Fiorito[1,*]

1 [1]Department of Veterinary Medicine and Animal Production, University of Naples Federico II, Naples, Italy; luca.delsorbo2@studenti.unina.it (L.D.S.), mariamichela.salvatore@unina.it (M.M.S.)

2 Department of Environmental, Biological and Pharmaceutical Sciences and Technologies, University of Campania Luigi Vanvitelli, Caserta, Italy; clementina.acconcia@unicampania.it (C.A.), luigi.russo2@unicampania.it (L.R.)

3 Istituto Zooprofilattico Sperimentale del Mezzogiorno, Portici (Naples), Italy;

4 Department of Veterinary Medicine, University of Bari, Valenzano (Bari), Italy; violetta.vasinioti@uniba.it (V.V.); mariastella.lucente@uniba.it (M.S.L.), annamaria.pratelli@uniba.it (A.P.)

5 College of Life Sciences, Hebei University, Baoding, 071002, China; lzhu3596@163.com (L.Z.)

6 Department of Chemical Science, University of Naples Federico II, Naples, Italy. andolfi@unina.it (A.A.)

[$]These authors contributed equally to this work.

* Correspondence: filomena.fiorito@unina.it (F.F.), giovanna.fusco@izsmportici.it (G.F.), rosa.iacovino@unicampania.it (R.I.)

Table S1. Schematic representation of the residues of the bovine AhR receptor involved in interactions with the inhibitor CH223191, as determined by docking calculations. The table lists the specific residues and their corresponding interactions, including hydrogen bonds, hydrophobic contacts, and other interactions that contribute to the binding affinity of CH223191 to the receptor.

INDEX	HYDROPHOBIC INTERACTIONS	HYDROGEN BONDS	Π - STACKING	Π – CATION INTERACTIONS
1	288 THR	345 SER	294 PHE	290 HIS
2	294 PHE	382 GLN		
3	307 LEU			
4	314 LEU			
5	323 PHE			
6	348 ILE			
7	350 PHE			
8	352 LEU			
9	366 ALA			
10	378 ILE			
11	380 ALA			

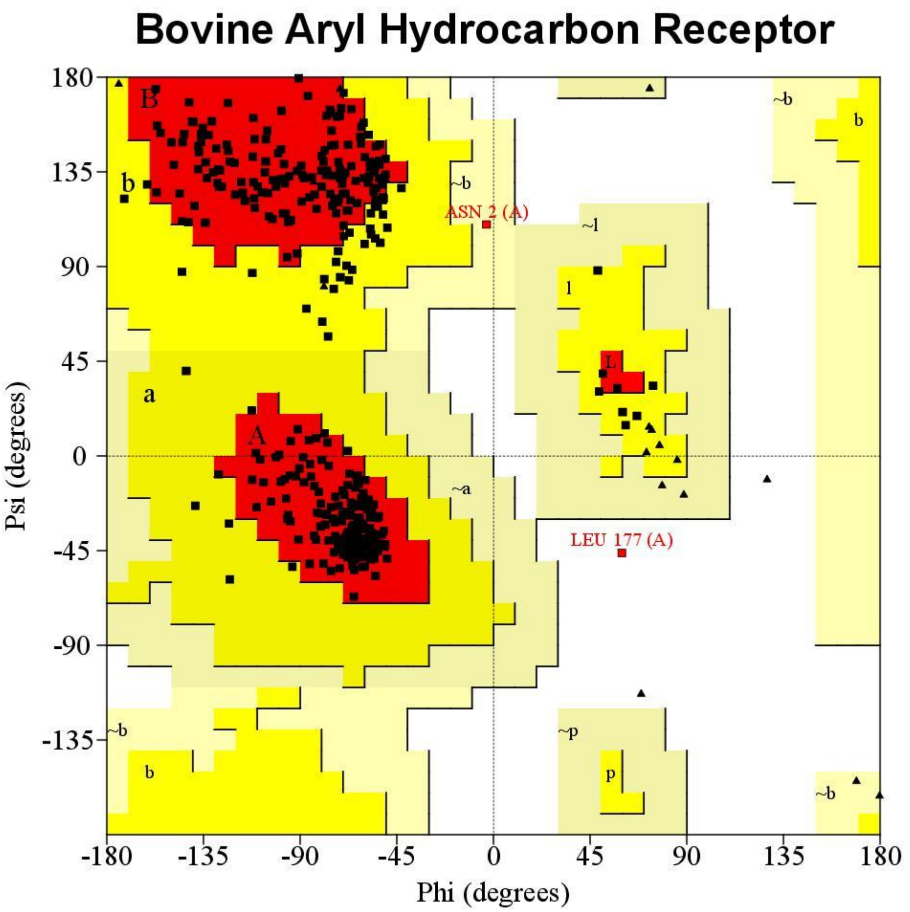

**Residues in most favoured regions**	**87.3 %**
**Residues in additional allowed regions**	**12.1%**
**Residues in generously allowed regions**	**0.3%**
**Residues in disallowed regions**	**0.3%**

Figure S1. Structural analysis of the three-dimensional model of the bovine Aryl Hydrocarbon Receptor (bAhR). The Ramachandran plot illustrates the conformational angles of the structural model, which was generated using AlphaFold, highlighting the distribution of dihedral angles and assessing the stereochemical quality of the predicted protein structure.

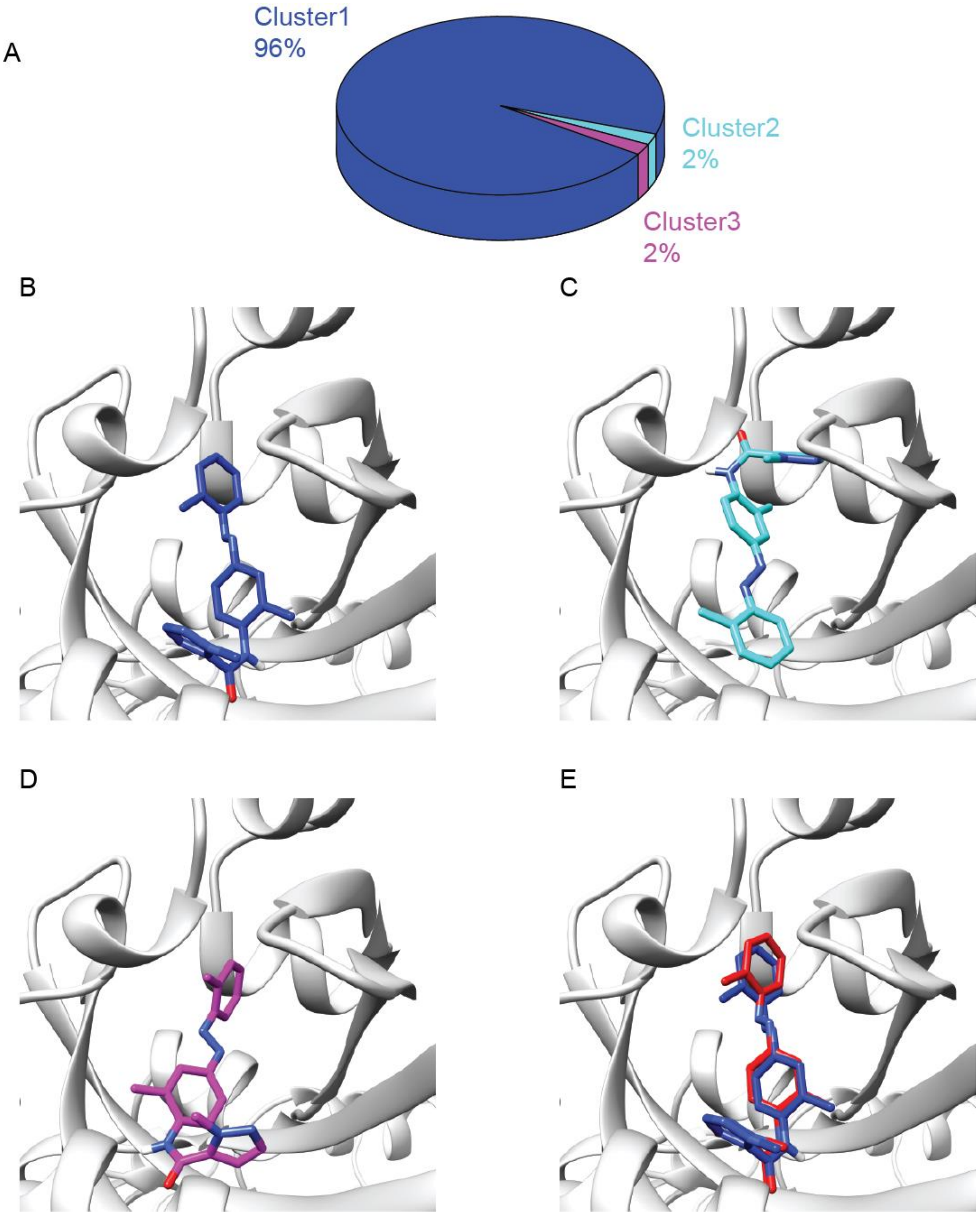

Figure S2. Molecular Docking studies of the bovine Aryl Hydrocarbon Receptor (bAhR) obtained by AutoDock 4.0 program. (A) Cluster analysis of the final ensemble obtained for the bAhR/ CH223191 complex. (B, C, D) The representative structure from cluster 1 (blue) (B), cluster 2 (cyan) (C) and cluster 3 (magenta) (D) is reported. The ligand and the receptor are illustrated as stick and light-grey ribbon drawing representation, respectively. (E) Comparison of the representative AutoDock 4.0 structure of the bAhR/ CH223191 complex (cluster 1) (blue) with that obtained using Dockthor software (red).
